# The effect of time-varying capacity utilization on 14-day in-hospital mortality: a retrospective longitudinal study in Swiss general hospitals

**DOI:** 10.1186/s12913-022-08950-y

**Published:** 2022-12-19

**Authors:** Narayan Sharma, Giusi Moffa, René Schwendimann, Olga Endrich, Dietmar Ausserhofer, Michael Simon

**Affiliations:** 1 Department Public Health (DPH), Institute of Nursing Science (INS), University of Basel, Bernoullistrasse 28, 4056 Basel, Switzerland; 2 Department of Mathematics and Computer Science, University of Basel, Basel, Switzerland; 3 Patient Safety Office, University Hospital Basel, Basel, Switzerland; 4 Directorate of Medicine, Inselspital University Hospital Bern, Bern, Switzerland; 5 College of Health Care-Professions Claudiana, Bozen, Italy

**Keywords:** Causal effect, Time-varying covariates, Capacity utilization, In-hospital mortality

## Abstract

**Background:**

High bed-occupancy (capacity utilization) rates are commonly thought to increase in-hospital mortality; however, little evidence supports a causal relationship between the two. This observational study aimed to assess three time-varying covariates—capacity utilization, patient turnover and clinical complexity level— and to estimate causal effect of time-varying high capacity utilization on 14 day in-hospital mortality.

**Methods:**

This retrospective population-based analysis was based on routine administrative data (*n* = 1,152,506 inpatient cases) of 102 Swiss general hospitals. Considering the longitudinal nature of the problem from available literature and expert knowledge, we represented the underlying data generating mechanism as a directed acyclic graph. To adjust for patient turnover and patient clinical complexity levels as time-varying confounders, we fitted a marginal structure model (MSM) that used inverse probability of treatment weights (IPTWs) for high and low capacity utilization. We also adjusted for patient age and sex, weekdays-vs-weekend, comorbidity weight, and hospital type.

**Results:**

For each participating hospital, our analyses evaluated the ≥85th percentile as a threshold for high capacity utilization for the higher risk of mortality. The mean bed-occupancy threshold was 83.1% (SD 8.6) across hospitals and ranged from 42.1 to 95.9% between hospitals. For each additional day of exposure to high capacity utilization, our MSM incorporating IPTWs showed a 2% increase in the odds of 14-day in-hospital mortality (OR 1.02, 95% CI: 1.01 to 1.03).

**Conclusions:**

Exposure to high capacity utilization increases the mortality risk of inpatients. Accurate monitoring of capacity utilization and flexible human resource planning are key strategies for hospitals to lower the exposure to high capacity utilization.

**Supplementary Information:**

The online version contains supplementary material available at 10.1186/s12913-022-08950-y.

## Background

Several observational studies have linked hospital bed-occupancy (capacity utilization) with in-hospital mortality [1–3]. While those studies account for numerous factors, they also acknowledge that the associations they show do not indicate causality [1, 2]. Logically, though, an unexpected rise in care demand (high patient volume, turnover, and case severity) could exceed a hospital’s human resource capacities on certain days (e.g., on weekends). Such situations would delay treatment for some patients and limit early recognition of clinical deterioration in others. Both cases contribute to adverse patient outcomes [3]. The link between capacity utilization and in-hospital mortality warrants further research due to the time-varying exposure of care demand [4]. Thus, the possible causal link between time-varying predictors and the outcome (e.g., in-hospital mortality) might require flexible care supply for safer hospitals in general.

The effect of a time-varying exposure is often confounded by time-varying variables [5]. One example of a confounder is daily patient flow, i.e., daily admission and discharge of patients. The confounder could affect outcomes such as mortality directly and also by influencing patient exposure to health care services (e.g., capacity utilization) at each measurement point. Over time, if the analysis does not account for this influence, it will distort the association between exposure and outcome. That is, the day-one exposure affects the value of day-two confounders and so on. In the language of causality, this is called exposure- or treatment-confounder feedback (TCF) [6]. In a hospital setting, the extent of daily capacity utilization may be influenced both by daily patient flow and disease severity; further, today’s capacity utilization might influence tomorrow’s patient flow. TCF induces a bias in traditional regression methods; as a result, they cannot control for time-varying factors that arise along the causal pathway between earlier exposure and later outcomes [5]. Neither the logistic regression used by Kuntz et al. nor the Poisson regression used by Madsen et al. attempted to identify and correct for TCF before assessing the association between bed-occupancy and in-hospital mortality [2, 3]. Estimating causal effects with time-varying variables requires Robins’ generalized methods [7, 8]. The most popular of these is the inverse probability of treatment weighting (IPTW) for marginal structural models (MSMs) [9].

To our knowledge, the effects of capacity utilization on in-hospital mortality have seldom been investigated using the causal inference framework and causal language. Previous studies aggregated bed-occupancy rates into monthly or annual estimates at the hospital level to define each institution’s threshold (i.e., the critical point at which there is a high risk of in-hospital mortality). For instance, the Kuntz et al., study in 83 German hospitals showed a threshold/safety tipping point at 92.5% of capacity utilization, after which the risk of in-hospital mortality increased significantly [3]. Yet, using a single threshold of capacity utilization for high risk of patients death may not be appropriate for a heterogeneous group of hospitals in the Swiss context [10]. Moreover, there is a substantial variation in patient care demand and supply (e.g., staffing) in Swiss hospitals due to the choice of complementary plans over basic healthcare plans, the Swiss Diagnosis-related groups (DRG), regulations in the 26 Swiss cantons and the size of the hospitals [11, 12]. Additionally, these findings do not support a causal interpretation, since no potential TCF was considered. To address these limitations, this study aimed to evaluate the causal effect of capacity utilization on 14-day in-hospital mortality consistent with directed acyclic graphs (DAGs) description of the data generating mechanism and the corresponding MSM/IPTW-based estimates.

## Methods

### Design, settings and participants

This is a retrospective longitudinal observational study using patient data routinely submitted to the Swiss Federal Statistics Office. As stipulated by article 22 of the Swiss Federal Act on Data Protection, the statistics office provided anonymized data on all Swiss hospital inpatients from 2012 to 2017. The statistics office classifies general hospitals into five types: university hospitals, tertiary care hospitals, large basic hospitals, medium basic hospitals and small basic hospitals. Each institution’s classification is based on the number of cases treated per year and/or a special hospital score assigned by the Swiss Medical Association [12, 13].

To comply with Swiss data protection regulations, we took only one-year annual patient population dataset; to reduce between-hospital heterogeneity, we included only general (acute care) hospitals. For the final models, we excluded patients admitted before the study year and over the last 2 weeks of the year, as it was impossible to link observations across the calendar year and to maintain consistency of maximum of 14 days of exposures and outcomes for each patient. Additional file 1 provides a flow diagram depicting our inpatient case selection process for analysis.

### Dataset and variables

The dataset included variables, like age in five-year categories, sex, hospitals/types, admission and discharge dates, diagnosis codes, in-hospital mortality. Additionally, individual-level variables Elixhauser index/Swiss comorbidity weights [14] and the dummy variable for weekends were computed from diagnosis codes and date of admission and discharge respectively.

Given the longitudinal nature of the problem, variables that change over time (e.g., daily capacity utilization, daily patient turnover and daily patient clinical complexity level (PCCL) were derived from all patients, on each of the days for the study year as time-varying exposures and confounders at the hospital level (Additional file 1, table S1). For a fair real-world comparison, daily patient turnover and capacity utilization were computed as percentages from the day of the study year with the most admitted patients for each hospital. Daily disease severity was computed as PCCL value per hospital per day [10]. PCCL is a measure of the cumulative effect of a patient’s comorbidities and/or complications for each episode of care. Values range from 0 (no complexity) to 4 (very severe complexity) [15]. The outcome of interest was 14-day in-hospital mortality, i.e., all deaths occurring during inpatients’ first 14 days in the hospital. We took 14 days of exposures and outcomes as more than 94% of all annual inpatients were discharged within the time frame. We considered staffing as an unmeasured variable as it was not available in our dataset.

### Treatment-exposure strategy

Our main exposure is represented by a binary variable indicating the level of capacity utilization as above or below a critical threshold of mortality risk. We derived the threshold from the annual distribution of capacity utilization for each hospital. Identifying a critical cut-off relevant to a particular outcome (mortality) is challenging. Some studies have found that a capacity utilization above 80–90% will lead to increases in infection risk, serious medical errors and mortality [2, 16, 17]. The study from Kuntz et al. [3] considered a safety tipping point at 92.5% of bed occupancy.

Our study included a heterogeneous group of 102 general hospitals whose capacity utilization varies throughout the year [10]. To explore the distribution of capacity utilization we used violin plots, with the 85th percentile as a breakpoint for each hospital. To evaluate the robustness of this approach we implemented sensitivity analyses using cut-offs bracketing the 85th percentile and computed the effect for each model. This shows that each hospital has a specific threshold of high risk to mortality at the 85th percentile (Additional file 1, table S2 and S3) and each hospital is unique in its capacity utilization distribution reflecting the heterogeneity of Swiss hospitals. From the daily binary indicator of exposure above the high-risk threshold of capacity utilization, we derived cumulative counts of days with above the threshold exposure, over 14 days, and used this in the models, as it accounts for high exposure and counts of past high exposures [18, 19].

### Exploring relationships between time-varying variables through DAGs

Causal DAGs are mathematically grounded [20] graphical representations of data-generating mechanisms. Additionally, thanks to graphical criteria based on the rules of d-separation, the structure of a DAG helps researchers identify and evaluate each variable’s role, i.e., as a mediator, collider or confounder [6]. Thus, when we wish to assess an exposure’s causal effect (if any) on an outcome, we can depict the available evidence as a DAG [6], which explicitly describes both the model and its underlying assumptions [21, 22].

We started the development of a causal DAG after this study’s conception. Mainly, we were interested in the relationship between capacity utilization and in-hospital mortality, while accounting for hospital and individual-level factors that may influence those relationships. We used two levels of capacity utilization as exposure, 14-day in-hospital mortality as the outcome and patient turnover and PCCL as the main time-varying confounders. To simplify the relationships shown in the DAG and to focus on the main concept we assumed no measurement error and no other confounding factors.

Blocking all back-door (i.e., non-causal) paths [23] between exposures and outcomes, allows adjusting for confounding and estimating the causal effect of an exposure on the targeted outcome. To construct a DAG that adequately captures the current knowledge about the process we used DAGitty [24], with several iterative steps to synthesize evidence by adding time-varying covariates, individual covariates and unmeasured variables. Additional file 1 provides the process of building causal DAGs for time-varying variables.

**Fig. 1:**
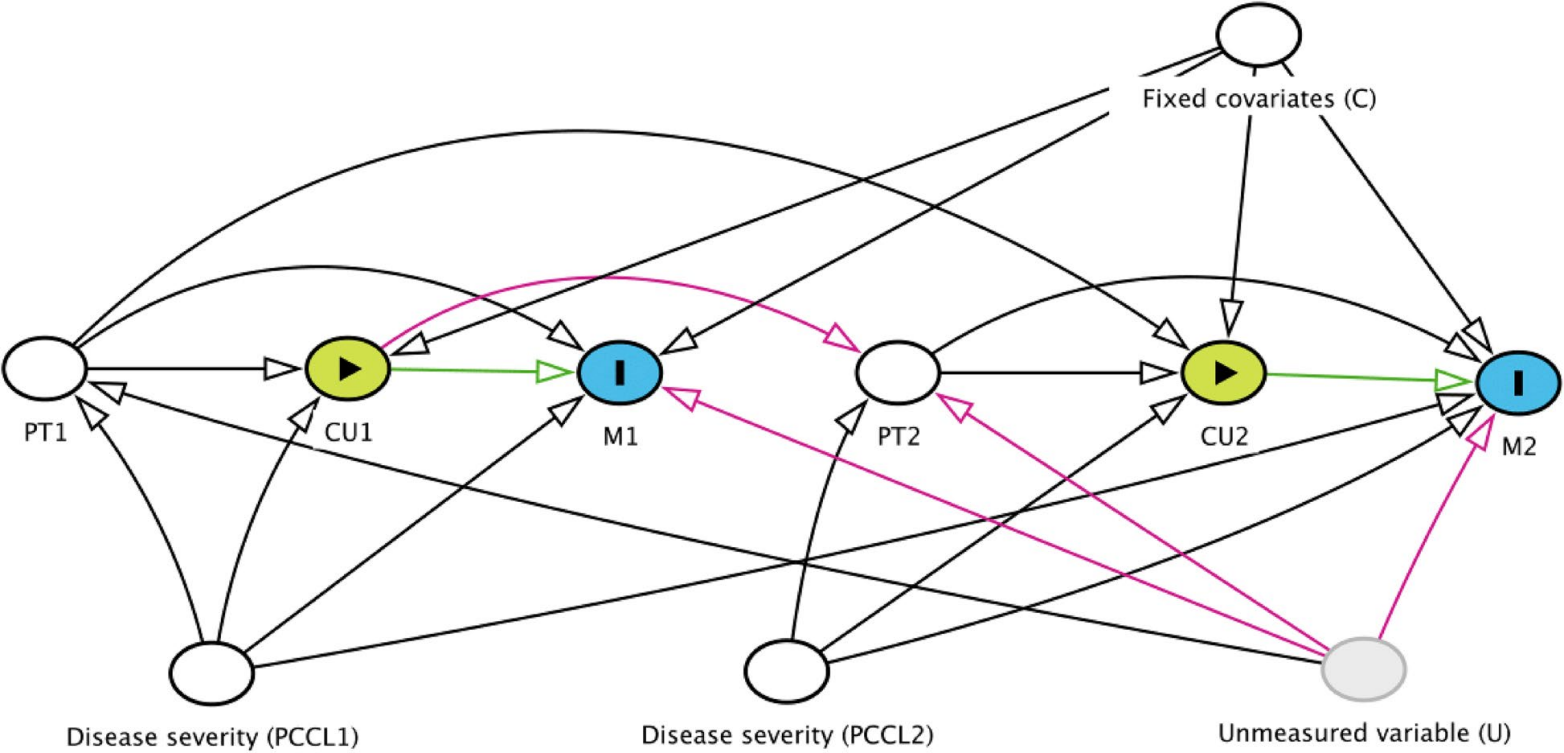
Causal DAG for time-varying exposure, time-varying confounders and outcomes at day one and day two. Time-varying exposures are capacity utilization (CU1, CU2), time-varying confounders are patient turnover (PT1, PT2), patient clinical complexity level (PCCL1, PCCL2) and outcome mortality (M1, M2) at day one and day two with other fixed covariates (C) (e.g., age, sex, comorbidity weights) and unmeasured variables (U) (e.g., staffing). The arrows are limited to prevent overcrowding, main issue remained same if we add more arrows and to avoid complexity in DAG, we consider single unmeasured variable. The green path represents a causal path (open), the black path an adjusted (blocked confounder) path and the red path the biasing path as per DAGitty

One underlying assumption is that the same graphical structure will repeat across various time points. Therefore, to simplify the visual representation, the final DAG in Fig. 1 shows only the connection between variables for the first 2 days in the two-week observation period. Including any unmeasured variables not captured in our dataset—e.g. staffing—in the DAG shows how that variable might affect both exposure and outcomes, e.g., staffing could be linked with patient turnover [25] and in-hospital mortality [26]. Day-one exposure (to capacity utilization) affects the time-varying confounder (patient turnover) on day two. We also need to acknowledge an unmeasured variable that affects both patient turnover and 14-day mortality forming TCF (Fig. 1). Therefore, traditional methods (e.g., stratification, outcome regression) are bound to produce biased estimates of the effect of capacity utilization on mortality [6, 27, 28].

### Statistical analysis

We reported the study population’s descriptive statistics overall and separately for all patients with 14-day mortality. Additionally, we described the daily distribution of each time-varying variable (capacity utilization, patient turnover and PCCL) via medians, interquartile ranges (IQRs), and minimum-maximum (Min-Max) per hospital and by hospital type.

In preparation for the statistical evaluation, we used the raw data to derive the time-varying and outcome variables in a format suitable for the intended longitudinal analysis. Each case was followed up for a maximum of 14 days. Where patients left or died within 14 days, their time-varying data were included respectively until discharge or death. Distributions of total exposure days for all study samples were explored for each hospital type [29].

We evaluated the suspected causative role of capacity utilization on in-hospital mortality based on a DAG describing the hypothesized mechanism over time (Fig. 1). To estimate the higher risk of death, we evaluated the daily capacity utilization equal to or above the 85th percentile for each hospital in the study year. Additionally, we adjusted for time-fixed covariates (age, sex, hospital type, Elixhauser index/Swiss comorbidity weight, and weekend) that affected both exposure and outcomes. Finally, the models also accounted for any clustering of the observations at the hospital and patient levels.

To estimate a causal effect of exposure to high capacity utilization on in-hospital mortality we fit MSMs using IPTW [9]. MSMs capture the relationship between the exposure and the potential outcomes, involving parameters directly describing causal effects [6]. IPTW is a strategy that allows us to estimate them from observational data, by eliminating any treatment confounding feedback and making exposure groups comparable [6, 30]. For longitudinal analysis, IPTW can be derived for each observation by multiplying the weights evaluated at each time point; the resulting weighting is usually stabilized to improve the precision of the MSM [31]. Basically, IPTW has the effect of creating an unconfounded population for low-capacity utilization versus high-capacity utilization. Further, it eliminates treatment-confounder feedback, which sets it apart from other regression methods [32, 33]. All time-fixed covariates (comorbidity weights, hospital types, weekdays, age and sex) were adjusted in the exposure model to calculate the IPTW using R software’s “ipw” package [34].

Marginal structural modelling is flexible enough to handle diverse types of data. In our case, we used multivariable logistic regression, with mortality as the binary outcome. To estimate the effect of increased exposure by one or more days before the endpoint, our model used cumulative days of exposure to high capacity utilization. Models were fitted using the generalized estimating equation (GEE) approach in R’s “geepack” package [3, 35–37]. For completeness, we conducted the analysis with and without IPTW [38]. Finally, we derived odds ratios (with 95% CIs) for fourteen-day in-hospital mortality with cumulative exposure to high capacity utilization.

To complement our primary analysis, we conducted an alternative analysis of the total/short-term effect where we used the daily binary indicator of high capacity utilization as exposure instead of a cumulative measure [39]. Finally, to assess the extent of potential model misspecifications while using IPTW, we also considered an analysis with stabilized weights truncated at their 1st and 99th percentiles.

## Results

We analysed annual data collected over one calendar year on 1,152,506 inpatient cases in 102 Swiss general hospitals, excluding admissions from the study year’s final 14 days. Of these, 53.4% were female. One-fifth of admissions were to university hospitals; 36.6% of patients had positive Elixhauser comorbidity weighting scores. Ten percent were very clinically complex. The overall fourteen-day in-hospital mortality rate was 1.5% (16,998); the death rate was highest (2.3%) in small basic hospitals. Detailed characteristics of the study population, including totals and fourteen-day mortality rates, are shown in Table 1.

**Table 1:** General characteristics of the study population in Swiss hospitals

	Total study population	14-days mortality (%)
Total population	1,152,506	16,998 (1.5)
Male	536,763	9662 (1.8)
Female	615,743	7567 (1.2)
Age groups		
0–19 years	152,887	558 (0.4)
20–29 years	85,498	79 (0.1)
30–39 years	125,141	156 (0.1)
40–49 years	99,401	373 (0.4)
50–59 years	139,695	1159 (0.8)
60–69 years	163,193	2270 (1.4)
70–79 years	192,614	4121 (2.1)
80–89 years	156,026	5803 (3.7)
90+ years	38,051	2479 (6.5)
Hospital types		
University (level 1)	222,552	3638 (1.6)
Tertiary care (level 2)	688,637	10,591 (1.5)
Large basic (level 3)	105,809	1194 (1.1)
Medium basic (level 4)	118,705	1191 (1.0)
Small basic (level 5)	16,803	384 (2.3)
Length of hospital stay (mean (SD)), days	6.2 (8.5)	–
Elixhauser index (mean (SD))	1.41 (1.81)	3.14 (2.06)
Elixhauser index (Swiss comorbidity weighting score)		
< 0	197,262	630 (0.3)
= 0	532,629	1694 (0.3)
> 0 to < 5	76,495	754 (1.0)
≥ 5	345,501	13,641 (3.9)
Individual (PCCL)		
No clinical complexity (0)	705,437	3759 (0.5)
Mild clinical complexity (1)	16,933	65 (0.4)
Moderate clinical complexity (2)	130,866	1346 (1.0)
Severe clinical complexity (3)	174,576	3915 (2.2)
Very severe clinical complexity (4)	124,694	7913 (6.3)
Days of admission		
Mondays	216,990	2890 (1.3)
Tuesdays	204,425	2700 (1.3)
Wednesdays	199,349	2561 (1.3)
Thursdays	184,595	2538 (1.4)
Fridays	161,107	2591 (1.6)
Saturdays	86,680	1844 (2.1)
Sundays	99,360	1874 (1.9)
Admission during weekdays		
Weekdays	966,466	13,530 (1.4)
Weekends	186,040	3718 (2.0)

Individual Patient Clinical Complexity Level (PCCL) ranges from 0 to 4, No clinical complexity, Mild clinical complexity, Moderate clinical complexity, Severe clinical complexity, Very severe clinical complexity

Each of the time-varying covariates varied daily across all general hospitals. In University hospitals, while daily capacity utilization and PCCL value were highest, daily patient turnover was lowest. The daily distributions of time-varying variables—capacity utilization, patient turnover and patient clinical complexity level—are shown in Additional file 1, table S4.

### Treatment-exposure strategy and distribution

**Fig. 2:**
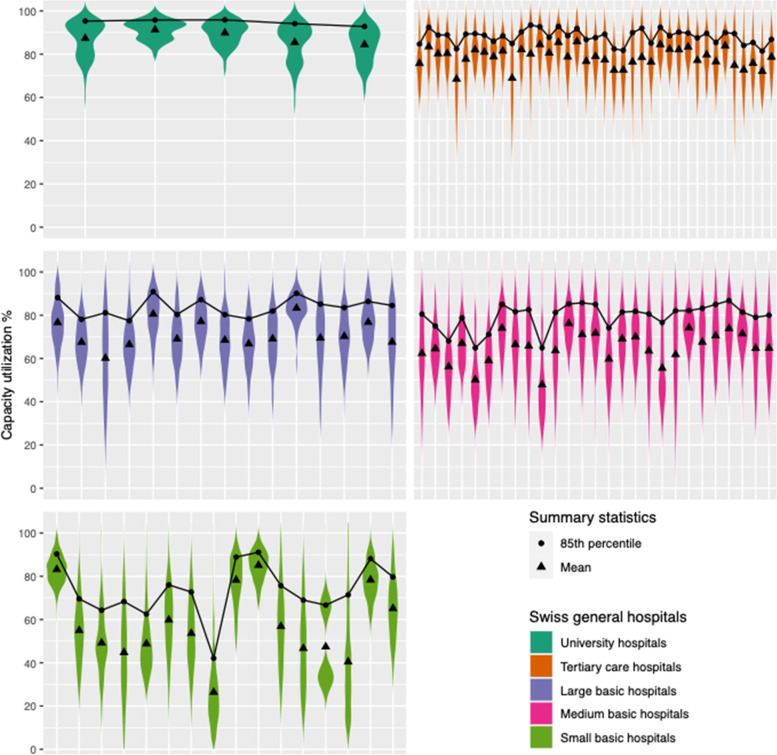
Violin plots showing the annual density distribution of daily capacity utilization (exposure) across 102 Swiss general hospitals. Violin plots across 102 Swiss general hospitals for a study year with 85th percentile and above showing higher risk of mortality and mean capacity utilization per hospital

Across general hospitals, the range of capacity utilization was distinct but broad—and much broader in small hospitals. On the hospital level, then, 85th percentile capacity utilization, which we considered a threshold for the high and low exposures [3], was unique for each institution (Additional file 1, table S2). Details of exposure to high capacity utilization—reaching the cut-offs evaluated per hospital type—are explored in Fig. 2 (85th Percentile and above). The mean threshold was 83.1% (SD 8.6) across hospitals. For university hospitals threshold ranged from 92.8 to 95.9%; for tertiary care hospitals they ranged from 81.4 to 93.5%; for large basic hospitals from 77.4 to 90.9%; for medium basic hospitals from 64.9 to 86.8%; and for small basic hospitals from 42.1 to 91.1%. The total study population was exposed to 6,867,658 hospital days, of which 1,279,021 (18.6%) included high capacity utilization. The robustness of the safety tipping point analysis, using different cut-offs of capacity utilization, is demonstrated in the (Additional file 1, table S3), showing the most fit at 85th percentile. The distribution of patient’s days for counterfactuals by hospital type is shown in the Additional file 1, table S5.

### Causal effect of time-varying capacity utilization on in-hospital mortality

The MSMs provide estimates of the causal effect of exposure to high capacity utilization on mortality (Table 2). The distribution of the computed stabilized weights was characterized by a median (IQR) of 0.99 (0.93 to 1.05), Min-Max equal to 0.17–18.8 and a mean of 1.00. The density distribution of the stabilized IPTW is shown in the (Additional file 1). One additional day of exposure to high capacity utilization increases the odds of 14-day in-hospital mortality by 2% (OR 1.02, 95% CI: 1.01 to 1.03).

**Table 2:** The adjusted effects of cumulative daily exposure to high capacity utilization on 14-day in-hospital mortality without and with IPTW (MSM)

	Without IPTW			With IPTW (MSM)		
	Estimate	*p*-value	Odds Ratio (95% CI)	Estimate	*p*-value	Causal Odds Ratio (95% CI)
Daily exposure to capacity utilization						
≥ 85th percentile per day	0.011	< 0.05	1.01 (1.00 to 1.02)	0.016	< 0.001	1.02 (1.01 to 1.03)
Other adjusted variables						
Weekend	0.091	< 0.001	1.10 (1.06 to 1.13)	0.088	< 0.001	1.09 (1.05 to 1.13)
Hospital types						
Tertiary care (level 2)	0.007	0.708	1.01 (0.97 to 1.05)	0.009	0.64	1.01 (0.97 to 1.05)
Large basic (level 3)	−0.149	< 0.001	0.86 (0.80 to 0.92)	− 0.141	< 0.001	0.87 (0.81 to 0.93)
Medium basic (level 4)	−0.114	< 0.001	0.89 (0.83 to 0.95)	−0.118	< 0.001	0.89 (0.83 to 0.95)
Small basic (level 5)	0.231	< 0.001	1.26 (1.13 to 1.40)	0.523	< 0.001	1.69 (1.48 to 1.92)
Elixhauser index (Swiss Comorbidity weights)						
= 0	1.090	< 0.001	2.97 (2.71 to 3.25)	1.090	< 0.001	2.97 (2.71 to 3.26)
> 0 to < 5	0.847	< 0.001	2.33 (2.10 to 2.60)	0.865	< 0.001	2.38 (2.13 to 2.65)
≥ 5	2.060	< 0.001	7.83 (7.22 to 8.49)	2.080	< 0.001	8.02 (7.39 to 8.71)
Age in 5 years	0.028	< 0.001	1.03 (1.03 to 1.03)	0.028	< 0.001	1.03 (1.03 to 1.03)
Female	−0.267	< 0.001	0.76 (0.74 to 0.79)	−0.270	< 0.001	0.76 (0.74 to 0.79)

Inverse Probability of Treatment/exposure Weight (IPTW) of capacity utilization ≥85th percentile for daily-varying confounders, patient turnover and PCCL (Patient Clinical Complexity Level). The reference categories are (hospital types: university hospitals, Swiss comorbidity weights: < 0). The age groups (five-year) are converted into numeric. Clustering of observations by hospital and patient is accounted for both models

For comparison, we also report odds ratios from the same multivariable logistic model as for the MSM—still using GEEs but without IPTW; therefore, this model is not adjusted for time-varying confounders. Using this model, an additional day of exposure to high capacity utilization was associated with only a 1% increase in the odds of 14-day in-hospital mortality (OR 1.01, 95% CI: 1.00 to 1.02).

Our analysis also highlights the odds of dying are 9% higher during weekends than on weekdays, and the odds of dying are higher for higher comorbidity scores. To document the heterogeneity among hospital types, the results indicate that the odds of dying are considerably higher in small basic hospitals and lower in medium and large basic hospitals than in university hospitals. And, the alternative analysis of the total/short term effect of high capacity utilization yielded 10% higher odds of 14-day mortality (Additional file 1, table S6). As expected, truncating at 1% of the IPTW data resulted in a slightly reduced effect but greater precision (Additional file 1, table S7).

## Discussion

This observational study examined the causal effect of time-varying capacity utilization on 14-day in-hospital mortality using data from 102 Swiss general hospitals. With an increase of 1 day in the cumulative number of days for which capacity utilization was high, there was a 2% increase in the odds of 14-day mortality. The effect size we found is a slightly increased effect with respect to that obtained via GEE without IPTW. Using IPTW to fit a MSM is expected to produce estimates which are adjusted for all time-varying and time-fixed confounders we identified in our DAG, therefore quite possibly closer to the real causal effect.

Exploring the distribution of the study hospitals’ capacity utilization revealed that each hospital had a different threshold for increased risk of patient death, i.e., confirming the heterogeneity of Swiss hospitals. Thus, capacity utilization might depend on each hospital’s size, available services and resources and particularly on hospitals’ management and planning. Madsen et al.’s 2014 study in 72 Danish hospitals used 80–85% for high bed occupancy, correlating this with a 9% increase in mortality [2]. Another study in 83 German hospitals placed the tipping point at 92.5% [3], showing one in seven deaths was possibly related to high occupancy. As these studies took bed occupancy at the time of admission and did not trace cumulative exposure over each hospital stay, their effects appear larger than they are. Moreover, they considered only one specific cut-off value for all studied hospitals. Therefore, they may not have adequately captured the threshold of inter-hospital variations of capacity utilization exposure, which might be of great interest for managers and policymakers.

This is a large-scale study, adjusting for multiple factors and accounting for clustering of observations by hospital and patients, directed at estimating the causal effect of capacity utilization on mortality. For instance, we also observed a weekend effect in Swiss hospitals and this may also have been influenced by staffing patterns [40]. Furthermore, the odds of dying were also higher for patients with higher comorbidity weighting scores [14]. These scores showed results similar to those of earlier studies in Canada and the US [41, 42].

As noted, to eliminate the effect of TCF, we computed IPTW for each case. This required including and evaluating time-varying confounders, e.g., daily patient turnover, daily PCCL value, alongside time-fixed variables. To our knowledge, this is the first use of a *G-method* [6] (e.g., fitting MSMs with IPTW [32]) to assess the causal effect of capacity utilization on in-hospital mortality.

Methodologically, then, this study differs in one major way from others that have used traditional methods of risk adjustment [17], it is the first to adjust for the bias of TCF [2, 43]. Further, DAGs allowed us to explore the qualitative relationships that link unmeasured variables such as staffing levels [26, 44] with both confounders and outcomes. A study in English general hospitals [26] showed a 3% increase in in-hospital mortality among patients cared for by fewer nursing staff (RNs and nursing assistants). To visualize the role of TCF, we assumed staffing as an unmeasured variable in our DAG. Even though the staffing variable was not included in our dataset, we believe IPTW-informed MSMs have corrected our estimates (e.g., staffing will impact patient turnover).

From a hospital-managerial perspective, uncovering substantial changes in capacity utilization over time calls for accurate monitoring of capacity utilization and its distribution. As variation in daily capacity utilization also changes the required resources/staff, e.g., the differences between workday and weekend workloads. Volatile capacity utilization lowers the critical point in small hospitals. For instance, in small basic hospitals, dynamic capacity utilization might even partly explain why their odds of inpatient mortality are higher than in university hospitals, even though they have lower average capacity utilization. A possible reason of low capacity utilization threshold in small hospitals might be due to the shortage of staffing [2], as induced by Swiss DRG for reducing cost [45], however, there might be negative consequences, when patients load doubles on some days of the year. The causal effect is likely driven by the variation of care demand and supply including factors like patient turnover and patient complexity in each hospital and their daily staffing patterns. Another consideration for future research could be to investigate exposure patterns of high capacity utilization during the early (e.g. < 5 days) or late hospitalization period.

This study had certain limitations. Firstly, Swiss data protection regulations prevented us from linking patient data from 1 year to another and unit-level analysis was not possible because of the lack of data about patients transferred between units. Therefore, we were also unable to construct full datasets for patients admitted over the entire study year and this might have somehow influenced our causal estimation. Moreover, we couldn’t trace variation of PCCL and Elixhauser comorbidities during the stay as ICD10 codes were reported for the total hospital stay. However, daily PCCL values per hospital were used as a time-varying confounder. Secondly, certain assumptions [31, 32] of causal inferences applied to our study. For example, although we considered including unmeasured variables (e.g., staffing level data or a proxy variable which was not available on a daily basis for all general hospitals) in our DAG our results remain hostage to the ignorability assumption [31, 46]. For instance, patients’ previous experiences with hospital services or that discharge may constitute a competing risk due to patient selection might challenge the study’s causal claims [3]. Another critical assumption was the positivity [31, 46], which we tried to address by using the 85th percentile (of each hospital’s highest occupancy) as a threshold of high exposure across all hospitals, rather than 85% of full capacity utilization. However, in some cases, a very short length of stay could either have only high exposure or only low exposure. The third important assumption was that our chosen IPTW model is correctly specified. As widespread weights may be an indication of misspecifications [46] we verified that the average IPTW was 1.0 and observed that min-max values were not particularly extreme.

## Conclusion

This observational study aimed to evaluate the causal effect of capacity utilization on 14-day in-hospital mortality in Swiss general hospitals. Using literature and expert knowledge, DAGs allowed us to determine time-varying exposure, confounders and fixed covariates. Our analyses using MSM with IPTW indicated that a one-day increase in cumulative exposure to high capacity utilization caused a 2% increase in the odds of 14-day in-hospital mortality in Swiss general hospitals. The mortality risk threshold varied across hospitals, depending on each institution’s distribution of capacity utilization throughout the year. Some hospitals’ widely distributed capacity utilization might have impaired their responsiveness to changing demands, possibly resulting in adverse outcomes including mortality. Finally, hospital managers need both to understand the risk of high capacity utilization and to know when they reach their thresholds to reduce the volatility of capacity utilization and ensure that resources safely meet daily care demands.

## Supplementary Information

**Additional file 1: Figure F1.** Flow diagram of the study participants from the study year for the analysis. **Table S1.** Description of the study variables. **Table S2.** Capacity utilization (%) at different percentile of annual distribution for each of the hospital. **Table S3.** Sensitivity analysis to evaluate robustness to different specifications of the capacity utilization tipping points in Swiss hospitals, splitting capacity utilization distribution at 75th, 80th, 85th, 90th, and 95th percentile. The table displays total/short-term effect of daily exposure on 14-day in-hospital mortality as estimated with generalized estimating equations (GEEs), for different values of the thresholds defining potential tipping points. **Figure F2.** DAGs A-D: Causal directed acyclic graphs (DAGs) showing causal effects of capacity utilization (CU) on in-hospital mortality (M). Note: the arrows are limited to prevent overcrowding. Green represents causal paths; black represents adjusted (blocked) paths; and pink represents biasing paths. A: Causal DAG for time-fixed confounders, exposure and outcome (PT1, PCCL1, CU1, M1: respectively, patient turnover, patient clinical complexity level, capacity utilization, and mortality at fixed-day/baseline). B: Causal DAG for time-varying confounders, exposure and outcome at day one (PT1, PCCL1, CU1, M1) and day two (PT2, PCC2, CU2, M2). C: Causal DAG for time-varying exposure, confounders and outcome at day one and day two with other fixed covariates (C) (age, sex, comorbidity weights, weeks). D: Causal DAG for time-varying exposure, confounders and outcome at day one and day two with other fixed covariates (C) and unmeasured variables (U) (e.g., leadership, staffing). Note: the arrows are limited to prevent overcrowding and main issue remained same if we add more arrows and to avoid complexity in DAG representation. **Table S4.** Daily distribution of time-varying variables capacity utilization, patient turnover and patient clinical complexity level per hospital by hospital type. **Table S5.** Patients’ treatment-exposure day distribution by hospital type. **Figure F3.** Density plot for inverse probability of treatment weight (IPTW) of capacity utilization with two time-varying confounders: patient turnover and patient clinical complexity level (PCCL). Inverse probability of treatment weight (IPTW) of capacity utilization with two time-varying confounders: patient turnover and patient clinical complexity level (PCCL) adjusting other fixed covariates. **Table S6.** Total/short-term effect of time-varying exposure (capacity utilization) on 14-day in-hospital mortality without and with IPTW. **Table S7.** Adjusted causal effect (total/short term and cumulative daily) of time-varying exposure (capacity utilization) on 14-day in-hospital mortality with truncation of the top and bottom 1% of IPTW.

## Data Availability

Upon application, the data that support the findings of this study are available from Federal Statistical Office (FSO), Switzerland.

## Abbreviations

DRGs: Diagnosis-Related Groups
DAGs: Directed Acyclic Graphs
GEE: Generalized Estimating Equation
IPTWs: Inverse Probability of Treatment Weights
MSM: Marginal Structure Model
PCCL: Patient Clinical Complexity Level
TCF: Treatment-Confounder Feedback
