# Supplementary material for: The effect of time-varying capacity utilization on 14-day in-hospital mortality: a retrospective longitudinal study in Swiss general hospitals
Source: BMC Health Serv Res. 2022 Dec 19;22:1551. doi: 10.1186/s12913-022-08950-y (PMC9764719; doi:10.1186/s12913-022-08950-y)
Supplement: Supplementary file 1 — Additional file 1: Figure F1. Flow diagram of the study participants from the study year for the analysis. Table S1. Description of the study variables. Table S2. Capacity utilization (%) at different percentile of annual distribution for each of the hospital. Table S3. Sensitivity analysis to evaluate robustness to different specifications of the capacity utilization tipping points in Swiss hospitals, splitting capacity utilization distribution at 75th, 80th, 85th, 90th, and 95th percentile. The table displays total/short-term effect of daily exposure on 14-day in-hospital mortality as estimated with generalized estimating equations (GEEs), for different values of the thresholds defining potential tipping points. Figure F2. DAGs A-D: Causal directed acyclic graphs (DAGs) showing causal effects of capacity utilization (CU) on in-hospital mortality (M). Note: the arrows are limited to prevent overcrowding. Green represents causal paths; black represents adjusted (blocked) paths; and pink represents biasing paths. A: Causal DAG for time-fixed confounders, exposure and outcome (PT1, PCCL1, CU1, M1: respectively, patient turnover, patient clinical complexity level, capacity utilization, and mortality at fixed-day/baseline). B: Causal DAG for time-varying confounders, exposure and outcome at day one (PT1, PCCL1, CU1, M1) and day two (PT2, PCC2, CU2, M2). C: Causal DAG for time-varying exposure, confounders and outcome at day one and day two with other fixed covariates (C) (age, sex, comorbidity weights, weeks). D: Causal DAG for time-varying exposure, confounders and outcome at day one and day two with other fixed covariates (C) and unmeasured variables (U) (e.g., leadership, staffing). Note: the arrows are limited to prevent overcrowding and main issue remained same if we add more arrows and to avoid complexity in DAG representation. Table S4. Daily distribution of time-varying variables capacity utilization, patient turnover and patient clinical complexity level per ho [file 12913_2022_8950_MOESM1_ESM.docx]

**Additional file 1. Supplementary Materials (figures and tables)**

**The effect of time-varying capacity utilization on 14-day in-hospital mortality: a retrospective longitudinal study in Swiss general hospitals**

**Figure F1.** Flow diagram of the study participants from the study year for the analysis

**
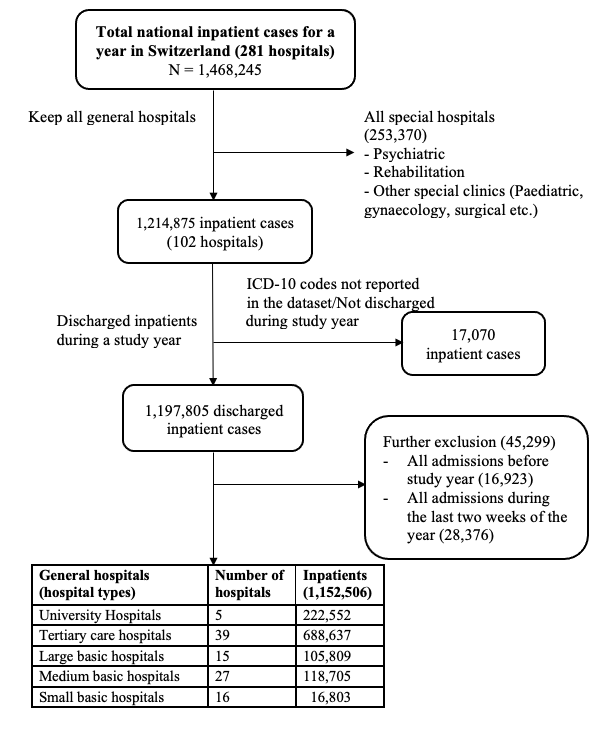
**

**Table S1.** Description of the study variables

| **Variables** | **Short description** |
| --- | --- |
| **Individual-level** |  |
| Case identifier | Unique anonymous numeric identifier for each case |
| Age | Age of patient in five-year groups |
| Sex | Patient’s sex (male or female) |
| Hospital identifier | Unique anonymous numeric identifier for each hospital |
| Hospital types | Hospital FSO classification code,  Five classifications: university hospitals, tertiary care hospitals, large basic hospitals, medium basic hospitals and small basic hospitals |
| Admission date | Patient’s date of hospital admission |
| Discharge date | Patient’s date of discharge from hospital |
| Primary Diagnosis | ICD–10 GM primary patient diagnosis code (up to 2) |
| Secondary diagnosis | ICD–10 GM secondary patient diagnosis code (up to 25) |
| Procedure code | CHOP code for procedure taken for the patient |
| 14–day in-hospital mortality | Death during the first 14 days of hospital stay (death or alive) |
| **Measured variables (individual level)** | |
| PCCL (0–4; no severity to very high severity) | Patient’s individual severity/complexity was measured using SwissDRG version 6 for batch grouping using diagnosis and procedural codes, using online grouping system[1] |
| Date related variables | Using the admission date of the study year, time-related variables were created as necessary for the analysis (e.g., Days of the week, weekdays vs weekend) |
| Elixhauser comorbidities  (31 comorbidities) | Number of Elixhauser comorbidities count (0, 1, 2,..15)  Number of Elixhauser comorbidities with Swiss comorbidity weights,[2] classified as (<0, 0, >0-4, >4) |
| **Measured variables (Hospital level)** | |
| Daily capacity utilization | Percentage of inpatients per day from the maximum daily observed inpatients (via admission and discharge dates) |
| Daily patient turnover | Percentage of admitted and discharged patients from the actual inpatients per day (via admission and discharge dates) |
| Daily PCCL | PCCL value for each individual was transformed to average daily PCCL value per day using admission and discharge dates |
| **Unmeasured variable** | **e.g., staffing level (nurses or doctors) might influence confounders, exposures and outcomes** |

**Source** Authors’ description of variables. **Notes** FSO Federal Statistics Office; ICD-10 GM International Classification of Diseases- version 10 German Modification; CHOP Swiss Operation Classification; PCCL Patient Clinical Complexity Level; Staffing level was considered as unmeasured variable (confounder) as daily staffing level was not available in our dataset

**Table S2.** Capacity utilization (%) at different percentile of annual distribution for each of the hospital

| **S.N** | **Anonymous hospital ID** | **Q75** | **Q80** | **Q85** | **Q90** | **Q95** |
| --- | --- | --- | --- | --- | --- | --- |
| 1 | 122 | 31.6 | 36.8 | 42.1 | 47.4 | 57.9 |
| 2 | 91 | 56.2 | 58.3 | 62.5 | 68.8 | 75.0 |
| 3 | 58 | 57.1 | 60.7 | 64.3 | 67.9 | 74.3 |
| 4 | 68 | 56.8 | 59.5 | 64.9 | 71.3 | 77.0 |
| 5 | 146 | 54.4 | 59.6 | 64.9 | 73.7 | 79.0 |
| 6 | 250 | 66.7 | 66.7 | 66.7 | 66.7 | 66.7 |
| 7 | 36 | 63.7 | 65.5 | 68.1 | 72.5 | 81.2 |
| 8 | 76 | 60.0 | 63.3 | 68.3 | 72.3 | 78.3 |
| 9 | 212 | 58.6 | 62.8 | 69.0 | 74.5 | 79.3 |
| 10 | 20 | 63.0 | 67.4 | 69.6 | 73.9 | 80.4 |
| 11 | 100 | 67.5 | 68.7 | 71.1 | 74.7 | 78.3 |
| 12 | 259 | 57.1 | 57.1 | 71.4 | 71.4 | 85.7 |
| 13 | 104 | 63.6 | 68.2 | 72.7 | 77.3 | 81.8 |
| 14 | 278 | 68.2 | 71.8 | 74.1 | 80.0 | 85.9 |
| 15 | 24 | 71.4 | 73.4 | 75.0 | 79.0 | 82.3 |
| 16 | 198 | 69.2 | 73.1 | 75.6 | 80.8 | 87.2 |
| 17 | 97 | 72.0 | 72.0 | 76.0 | 80.0 | 84.0 |
| 18 | 370 | 67.6 | 70.3 | 76.6 | 79.7 | 83.8 |
| 19 | 144 | 73.2 | 75.0 | 77.4 | 80.5 | 85.4 |
| 20 | 31 | 75.0 | 76.9 | 78.1 | 80.4 | 83.8 |
| 21 | 231 | 74.7 | 75.9 | 78.3 | 80.7 | 85.5 |
| 22 | 53 | 75.0 | 76.7 | 78.8 | 80.1 | 84.2 |
| 23 | 481 | 74.4 | 77.9 | 79.1 | 83.7 | 87.2 |
| 24 | 520 | 74.6 | 76.3 | 79.7 | 83.0 | 88.1 |
| 25 | 576 | 75.0 | 76.7 | 80.0 | 82.2 | 86.7 |
| 26 | 209 | 77.1 | 78.6 | 80.3 | 83.6 | 85.0 |
| 27 | 156 | 76.8 | 78.6 | 80.4 | 83.9 | 88.7 |
| 28 | 10 | 75.4 | 77.7 | 80.5 | 83.8 | 87.7 |
| 29 | 326 | 74.8 | 77.3 | 80.6 | 83.3 | 88.3 |
| 30 | 48 | 75.4 | 77.9 | 81.2 | 84.9 | 89.3 |
| 31 | 155 | 76.6 | 78.9 | 81.2 | 85.2 | 89.0 |
| 32 | 288 | 76.8 | 79.4 | 81.4 | 84.5 | 87.6 |
| 33 | 573 | 76.9 | 79.0 | 81.4 | 84.2 | 89.9 |
| 34 | 472 | 78.1 | 79.8 | 81.5 | 83.7 | 86.5 |
| 35 | 114 | 75.0 | 76.8 | 81.6 | 85.4 | 89.3 |
| 36 | 293 | 78.0 | 79.5 | 81.8 | 83.3 | 87.9 |
| 37 | 353 | 79.3 | 80.4 | 81.8 | 84.8 | 87.6 |
| 38 | 273 | 78.3 | 79.5 | 81.9 | 84.1 | 86.8 |
| 39 | 371 | 76.9 | 79.8 | 82.1 | 85.7 | 90.1 |
| 40 | 390 | 79.3 | 81.0 | 82.1 | 83.8 | 86.6 |
| 41 | 148 | 78.1 | 80.0 | 82.5 | 85.4 | 89.2 |
| 42 | 336 | 79.4 | 80.7 | 82.5 | 84.2 | 88.1 |
| 43 | 116 | 77.7 | 79.5 | 82.6 | 85.6 | 89.4 |
| 44 | 426 | 78.2 | 79.8 | 83.2 | 85.7 | 87.4 |
| 45 | 479 | 79.1 | 80.9 | 83.5 | 85.2 | 87.9 |
| 46 | 566 | 79.9 | 81.3 | 84.0 | 87.0 | 91.8 |
| 47 | 500 | 80.5 | 83.5 | 84.5 | 87.0 | 89.9 |
| 48 | 18 | 80.9 | 82.5 | 84.7 | 89.3 | 92.0 |
| 49 | 184 | 79.9 | 82.0 | 84.9 | 87.1 | 90.3 |
| 50 | 106 | 82.6 | 83.2 | 85.0 | 86.8 | 89.8 |
| 51 | 196 | 79.5 | 82.7 | 85.0 | 88.0 | 91.3 |
| 52 | 443 | 80.0 | 81.7 | 85.0 | 86.7 | 91.7 |
| 53 | 402 | 82.3 | 83.6 | 85.1 | 87.3 | 89.1 |
| 54 | 179 | 82.8 | 83.6 | 85.2 | 86.9 | 90.2 |
| 55 | 349 | 80.0 | 82.6 | 85.2 | 88.1 | 91.6 |
| 56 | 515 | 82.5 | 83.8 | 85.4 | 89.0 | 94.5 |
| 57 | 571 | 81.2 | 83.0 | 85.4 | 89.2 | 92.2 |
| 58 | 181 | 81.4 | 83.8 | 85.7 | 88.2 | 90.7 |
| 59 | 170 | 84.2 | 84.8 | 85.9 | 88.0 | 91.2 |
| 60 | 482 | 83.1 | 84.4 | 86.4 | 89.5 | 92.0 |
| 61 | 304 | 83.6 | 84.5 | 86.7 | 88.8 | 92.8 |
| 62 | 445 | 82.4 | 84.3 | 86.8 | 89.6 | 92.6 |
| 63 | 575 | 84.6 | 85.4 | 86.8 | 90.0 | 92.5 |
| 64 | 158 | 83.6 | 86.0 | 87.2 | 89.3 | 92.1 |
| 65 | 495 | 85.0 | 86.5 | 87.5 | 89.5 | 92.1 |
| 66 | 314 | 84.1 | 86.3 | 87.7 | 89.0 | 92.5 |
| 67 | 220 | 85.1 | 86.3 | 87.8 | 89.1 | 93.4 |
| 68 | 28 | 83.0 | 85.9 | 88.1 | 91.5 | 94.8 |
| 69 | 333 | 84.5 | 85.7 | 88.1 | 90.5 | 91.7 |
| 70 | 176 | 86.2 | 87.2 | 88.4 | 90.2 | 91.9 |
| 71 | 240 | 85.4 | 87.0 | 88.5 | 89.7 | 92.0 |
| 72 | 448 | 87.1 | 87.8 | 88.5 | 89.7 | 92.7 |
| 73 | 50 | 85.5 | 87.2 | 88.8 | 91.3 | 93.2 |
| 74 | 165 | 85.6 | 86.8 | 88.8 | 91.0 | 93.4 |
| 75 | 87 | 85.9 | 86.7 | 88.9 | 91.1 | 93.3 |
| 76 | 178 | 88.9 | 88.9 | 88.9 | 88.9 | 94.4 |
| 77 | 150 | 84.2 | 87.2 | 89.3 | 91.3 | 94.3 |
| 78 | 317 | 85.1 | 86.8 | 89.3 | 91.3 | 93.4 |
| 79 | 161 | 86.7 | 87.5 | 89.5 | 92.1 | 94.5 |
| 80 | 555 | 85.2 | 87.3 | 89.5 | 92.0 | 94.3 |
| 81 | 502 | 86.9 | 88.4 | 89.7 | 91.2 | 93.4 |
| 82 | 483 | 87.6 | 88.8 | 89.8 | 91.8 | 95.3 |
| 83 | 368 | 86.3 | 88.2 | 90.0 | 91.6 | 92.9 |
| 84 | 530 | 88.2 | 88.9 | 90.0 | 90.9 | 92.4 |
| 85 | 294 | 88.2 | 88.8 | 90.1 | 91.3 | 93.7 |
| 86 | 462 | 87.8 | 88.7 | 90.2 | 91.4 | 93.9 |
| 87 | 2 | 88.2 | 89.2 | 90.3 | 91.4 | 93.5 |
| 88 | 189 | 88.0 | 89.1 | 90.3 | 91.8 | 94.2 |
| 89 | 153 | 88.5 | 89.7 | 90.9 | 92.1 | 95.0 |
| 90 | 183 | 91.1 | 91.1 | 91.1 | 93.3 | 95.6 |
| 91 | 264 | 90.2 | 90.9 | 91.8 | 93.5 | 96.2 |
| 92 | 374 | 88.0 | 90.5 | 92.0 | 93.1 | 95.6 |
| 93 | 34 | 90.5 | 91.6 | 92.4 | 93.9 | 95.6 |
| 94 | 427 | 89.4 | 90.8 | 92.4 | 94.1 | 95.8 |
| 95 | 207 | 89.9 | 91.0 | 92.6 | 94.2 | 95.3 |
| 96 | 234 | 91.2 | 91.8 | 92.8 | 93.5 | 95.0 |
| 97 | 398 | 90.5 | 92.0 | 92.8 | 94.2 | 95.5 |
| 98 | 200 | 89.4 | 91.7 | 93.5 | 94.8 | 96.4 |
| 99 | 343 | 92.3 | 93.1 | 94.1 | 95.1 | 96.5 |
| 100 | 23 | 94.0 | 94.6 | 95.3 | 95.9 | 96.9 |
| 101 | 193 | 94.7 | 95.3 | 95.8 | 96.5 | 97.7 |
| 102 | 252 | 94.0 | 94.8 | 95.9 | 96.6 | 97.6 |

**Source** Authors’ analysis of Swiss hospitals data. **Notes** Q75, Q80, Q85, Q90, and Q95 are 75^th^, 80^th^, 85^th^, 90^th^, and 95^th^ percentiles of annual distribution. For example, for the last hospital in table S2, this hospital operates at 94% and 96% for 75^th^ and 85^th^ percentile, looking at table S4, the odds ratio is 1.04 and 1.08, so models suggest to operate this hospital below 94% of capacity utilization as possible. This applies to other hospitals accordingly.

**Table S3.** Sensitivity analysis to evaluate robustness to different specifications of the capacity utilization tipping points in Swiss hospitals, splitting capacity utilization distribution at 75th, 80th, 85th, 90th, and 95th percentile. The table displays total/short-term effect of daily exposure on 14-day in-hospital mortality as estimated with generalized estimating equations (GEEs), for different values of the thresholds defining potential tipping points.

| GEE glm models | Odds Ratio (95% CI) |
| --- | --- |
| 75^th^ Percentile split | 1.04 (1.01–1.08)* |
| 80^th^ Percentile split | 1.06 (1.02–1.10)** |
| **85^th^ Percentile split** | **1.08 (1.03–1.12)***** |
| 90^th^ Percentile split | 1.07 (1.03–1.13)** |
| 95^th^ Percentile split | 1.07 (1.01–1.14)* |

**Source** Authors’ analysis of Swiss hospitals data. **Notes** Signifiance codes: p <0.001, **p <0.01, *p <0.05. The effect estimate on each split was positive with similar effects at and above 85th percentile, indicating that our capacity utilization split was robust.

Time-fixed covariates (age, sex, hospital types, Elixhauser index-Swiss comorbidity weights, and weekdays) affecting both exposure and outcomes were adjusted in each model.

**Building causal DAGs for time-varying variables to estimate causal effect of capacity utilization on in-hospital mortality**

Our research team (epidemiologist/statistician, patient safety officer, physician, nurses and public health researcher) conceptualized the relationships based on the literature and/or expert knowledge. Additionally, background knowledge from previous studies/surveys/interviews in hospitals were considered along with training in causal inference methodology and causal diagrams.

The literature indicates a clear relationship between capacity utilization (exposure) and in-hospital mortality (outcome) [3, 4]. Problematically, though, capacity utilization and mortality could be confounded by disease severity (patient clinical complexity level (PCCL)) and patient turnover. I.e., these variables can open up backdoor paths to apparent causality. To block these paths, it is necessary to identify and adjust for confounders.

To explore potential confounding relationships, we used DAGs with time-fixed variables (Figure S1 A). DAGs may clarify relationships between selected variables in a cross-sectional study. They can also be used for time-varying studies such as this one, but multiple measurement points have to be included.

To explore time-varying variables and limit our DAGs’ complexity, we used two consecutive days as measurement points (day one (baseline): admission day; day two: the following day) (Figure S1 B). Assuming that the same relationships would apply at later time points, we drew paths from the confounders to exposure and outcome for each day of the patient’s stay, i.e., every day’s capacity utilization and mortality figures are influenced by daily patient turnover and daily PCCL values.

Additionally, day-one capacity utilization influences day-two patient turnover. For example, if capacity utilization is high on day one, the hospital may admit fewer and/or discharge more patients on day two; prospective admissions’ PCCL scores can also influence both admissions and discharges. Therefore, as Figure 2 B shows, we placed an arrow from CU1 to PT2 to indicate feedback between the treatment and the confounder, i.e., TCF. As our DAG considered measurements for all of our dataset’s potential confounders, we were able to estimate the hypothesized causal effect by implementing an adjusted analysis.

Again, the given DAG is a valid causal model only if it includes every potential confounder. To account for additional individual covariates (e.g., age, sex, Swiss comorbidity weights, weekday/weekend) we built a causal DAG (see Figure 2 C) including all additional time-fixed confounders for which adjustment was necessary. This step allowed us to estimate the total causal effect of capacity utilization on in-hospital mortality.

Additionally, we considered the possible relevance of unmeasured variables not included in our dataset (e.g., supportive leadership or staffing). These may also influence the measured variables, e.g., staffing may affect both patient turnover [5] and in-hospital mortality [6]. In this condition, feedback (TCF) between day-one capacity utilization and day-two patient turnover persists. That is, the unmeasured variables (U) leave an open back-door path (CU1-PT2-U-M1) (Figure 2 D). Thus, this condition could lead to bias via two pathways: 1. Adjusting for patient turnover (PT2) to block the confounding CU2–PT2–M2 path will open another (through CU1, PT2, U and M2) by conditioning on a collider; or 2. If we do not adjust for patient turnover (PT2) we leave a back door path open through CU2-PT2-M2.

Traditional methods (e.g., stratification, outcome regression) cannot adjust for both of these confounders at the same time [7-9]. Therefore, methods to handle time-varying variables and TCF are necessary. Robins’ G-methods are one way of dealing with this problem. In addition to G-computation[10] and g-estimation for Structural Nested Models [11], these include the use of inverse probability of treatment weighting (IPTW) for Marginal Structural Models (MSMs) [12].

**In IPTW**, confounding is adjusted by weighting equal to the inverse of the participant’s probability of receiving their exposure history at each visit, given the value of previous confounders [13]. **MSM** is a multi-step estimation procedure designed to control for the effect of confounding variables that change over time, and are affected by previous treatment [14]. Particularly MSM (IPTW fitted in one of the longitudinal methods i.e., GEE) models the relationship between the covariate and the putative cause (i.e., capacity utilization in our case). MSM exhibit balance on the covariates, a property that would also be expected under randomization. Balanced covariates cannot be confounders anymore as they are unrelated to treatment assignment, and it is through this balance property of IPTW for MSM addresses issues of bias due to observed time-varying confounders [15]. Example, time-varying confounders (patient turnover and PCCL) are balanced between high and low capacity utilization via IPTW for MSM. Therefore, they are no more confounders.

**Assumptions of causal inferences**

Inference of an exposure’s causality regarding an outcome relies on a set of *identifiability assumptions*, regardless large volume of data [7]. The three most common of these are 1) ignorability/exchangeability (no unmeasured confounding’s are further present), 2) positivity (any individual has a positive probability of receiving all values of the treatment/exposure) and 3) correct specification of the IPTW model [7, 15, 16].

**References (Building causal DAGs, MSM and assumptions)**

1. SwissDRG: Swiss Diagnosis Related Groups Version 8.0. In*.* Bern, Switzerland: SwissDRG AG; 2018.

2. Sharma N, Schwendimann R, Endrich O, Ausserhofer D, Simon M: Comparing Charlson and Elixhauser comorbidity indices with different weightings to predict in-hospital mortality: an analysis of national inpatient data. *BMC Health Serv Res* 2021, 21(13).

3. Boden D, Agarwal A, Hussain T, Martin S, Radford N, Riyat M, So K, Su Y, Turvey A, Whale C: Lowering levels of bed occupancy is associated with decreased inhospital mortality and improved performance on the 4-hour target in a UK District General Hospital. *Emergency Medicine Journal* 2016, 33(2):85-90.

4. Kuntz L, Mennicken R, Scholtes S: Stress on the ward: Evidence of safety tipping points in hospitals. *Management Science* 2014, 61(4):754-771.

5. Park SH, Blegen MA, Spetz J, Chapman SA, De Groot H: Patient turnover and the relationship between nurse staffing and patient outcomes. *Research in nursing & health* 2012, 35(3):277-288.

6. Griffiths P, Maruotti A, Saucedo AR, Redfern OC, Ball JE, Briggs J, Dall'Ora C, Schmidt PE, Smith GB: Nurse staffing, nursing assistants and hospital mortality: retrospective longitudinal cohort study. *BMJ quality & safety* 2019, 28(8):609-617.

7. Hernán MA, Robins JM: Causal inference: what if. Boca Raton: Chapman & Hall/CRC; 2020.

8. McGrath S, Lin V, Zhang Z, Petito LC, Logan RW, Hernán MA, Young JG: gfoRmula: An R Package for Estimating the Effects of Sustained Treatment Strategies via the Parametric g-formula. *Patterns* 2020:100008.

9. Hernán MA: How to estimate the effect of treatment duration on survival outcomes using observational data. *bmj* 2018, 360.

10. Robins J: A new approach to causal inference in mortality studies with a sustained exposure period—application to control of the healthy worker survivor effect. *Mathematical modelling* 1986, 7(9-12):1393-1512.

11. Vansteelandt S, Joffe M: Structural nested models and G-estimation: the partially realized promise. *Statistical Science* 2014, 29(4):707-731.

12. Robins JM: Marginal structural models versus structural nested models as tools for causal inference. In: *Statistical models in epidemiology, the environment, and clinical trials.* edn.: Springer; 2000: 95-133.

13. Mansournia MA, Etminan M, Danaei G, Kaufman JS, Collins G: Handling time varying confounding in observational research. *bmj* 2017, 359.

14. Williamson T, Ravani P: Marginal structural models in clinical research: when and how to use them? *Nephrology Dialysis Transplantation* 2017, 32(suppl_2):ii84-ii90.

15. Thoemmes F, Ong AD: A primer on inverse probability of treatment weighting and marginal structural models. *Emerging Adulthood* 2016, 4(1):40-59.

16. Using inverse probability of treatment weights & Marginal structural models to handle time-varying covariates [<https://rpubs.com/mbounthavong/IPTW_MSM_Tutorial>]

| ****  A |
| --- |
| ****  B |
|   C |
|  D |

**Figure F2** DAGs A-D: Causal directed acyclic graphs (DAGs) showing causal effects of capacity utilization (CU) on in-hospital mortality (M). Note: the arrows are limited to prevent overcrowding. Green represents causal paths; black represents adjusted (blocked) paths; and pink represents biasing paths.

A: Causal DAG for time-fixed confounders, exposure and outcome (PT1, PCCL1, CU1, M1: respectively, patient turnover, patient clinical complexity level, capacity utilization, and mortality at fixed-day/baseline).

B: Causal DAG for time-varying confounders, exposure and outcome at day one (PT1, PCCL1, CU1, M1) and day two (PT2, PCC2, CU2, M2).

C: Causal DAG for time-varying exposure, confounders and outcome at day one and day two with other fixed covariates (C) (age, sex, comorbidity weights, weeks).

D: Causal DAG for time-varying exposure, confounders and outcome at day one and day two with other fixed covariates (C) and unmeasured variables (U) (e.g., leadership, staffing).

Note: the arrows are limited to prevent overcrowding and main issue remained same if we add more arrows and to avoid complexity in DAG representation.

**Table S4.** Daily distribution of time-varying variables capacity utilization, patient turnover and patient clinical complexity level per hospital by hospital type

| Hospital Types | Number of hospitals | Capacity utilization (%) | | Patient turnover (%) | | Patient clinical complexity level (0-4) | |
| --- | --- | --- | --- | --- | --- | --- | --- |
|  | N | Median  (IQR) | Min–Max | Median  (IQR) | Min–Max | Median  (IQR) | Min–Max |
| University Hospitals | 5 | 89.3  (83.3–93.5) | 55.8–100 | 21.8  (17.1–29.4) | 5.7–38.7 | 2.06  (2.05–2.07) | 0.81–2.57 |
| Tertiary care hospitals | 39 | 79.5  (72.5–86) | 27.3–100 | 29.3  (23.9–34.1) | 2.7–54.6 | 1.78  (1.78–1.79) | 0.42–2.75 |
| Large basic hospitals | 15 | 72.3  (62.9–81.1) | 13.1–100 | 33.3  (25.9–39) | 0–75.4 | 1.46  (1.45–1.47) | 0.09–2.50 |
| Medium basic hospitals | 27 | 66.4  (55.1–76.5) | 5.9–100 | 34.5  (27.5–41.5) | 0–109.1 | 1.26  (1.25–1.27) | 0.00–2.93 |
| Small basic hospitals | 16 | 59.1  (41–76.7) | 1.7–100 | 21.4  (5.9–37) | 0–200 | 1.65  (1.63–1.67) | 0.00–4.00 |

Variables measured in Inter Quartile Range (IQR) and Minimum–Maximum (Min–Max) and Patient clinical complexity level (0-4) (respectively) i.e., No clinical complexity, Mild clinical complexity, Moderate clinical complexity, Severe clinical complexity, Very severe clinical complexity. Maximum utilization on hospital level was 100% on the day with the most patients admitted during the year

**Table S5.** Patients’ treatment-exposure day distribution by hospital type

| Parameters | Total exposure days | Exposure (capacity utilization) days for below and above tipping points (n (%)) | | SMD |
| --- | --- | --- | --- | --- |
|  |  | < 85^th^ percentile | ≥ 85th percentile |  |
| Total | 6,867,658 | 5,588,637 (81.4) | 1,279,021 (18.6) |  |
| Hospital types |  |  |  | 0.075 |
| University (level 1) | 1,474,338 | 1,216,182 (82.5) | 258,156 (17.5) |  |
| Tertiary care (level 2) | 4,073,652 | 3,326,103 (81.6) | 747,549 (18.4) |  |
| Large basic (level 3) | 579,174 | 463,261 (80.0) | 115,913 (20.0) |  |
| Medium basic (level 4) | 628,306 | 497,660 (79.2) | 130,646 (20.8) |  |
| Small basic (level 5) | 112,188 | 85,431 (76.1) | 26,757 (23.9) |  |

Standardized mean difference (SMD) between < 85^th^ percentile and ≥ 85^th^ percentile

**Figure F3.** Density plot for inverse probability of treatment weight (IPTW) of capacity utilization with two time-varying confounders: patient turnover and patient clinical complexity level (PCCL). Inverse probability of treatment weight (IPTW) of capacity utilization with two time-varying confounders: patient turnover and patient clinical complexity level (PCCL) adjusting other fixed covariates.

**Table S6.** Total/short-term effect of time-varying exposure (capacity utilization) on 14-day in-hospital mortality without and with IPTW

|  | Without IPTW | | | With IPTW | | |
| --- | --- | --- | --- | --- | --- | --- |
|  | Estimate | p value | Odds Ratio (95% CI) | Estimate | p value | Causal Odds Ratio (95% CI) |
| Daily exposure to capacity utilization | | | | | | |
| ≥85^th^ percentile per day | 0.074 | <0.001 | 1.08  (1.03 to 1.12) | 0.097 | <0.001 | 1.10  (1.06 to 1.15) |
| Other adjusted variables | | | | | | |
| Weekend | 0.108 | <0.001 | 1.11  (1.08 to 1.15) | 0.110 | <0.001 | 1.12  (1.08 to 1.16) |
| Hospital types |  |  |  |  |  |  |
| Tertiary care (level 2) | 0.007 | 0.712 | 1.01  (0.97 to 1.05) | 0.022 | 0.64 | 1.01  (0.97 to 1.05) |
| Large basic (level 3) | -0.149 | <0.001 | 0.86  (0.80 to 0.92) | -0.141 | <0.001 | 0.87  (0.81 to 0.93) |
| Medium basic (level 4) | -0.114 | <0.001 | 0.89  (0.83 to 0.95) | -0.118 | <0.001 | 0.89  (0.83 to 0.95) |
| Small basic (level 5) | 0.231 | <0.001 | 1.26  (1.13 to 1.40) | 0.525 | <0.001 | 1.69  (1.48 to 1.93) |
| Elixhauser index (Swiss Comorbidity weights) | | | | | | |
| =0 | 1.090 | <0.001 | 2.97  (2.71 to 3.25) | 1.090 | <0.001 | 2.97  (2.71 to 3.26) |
| >0 to <5 | 0.847 | <0.001 | 2.33  (2.10 to 2.59) | 0.865 | <0.001 | 2.38  (2.13 to 2.65) |
| ≥5 | 2.060 | <0.001 | 7.83  (7.22 to 8.49) | 2.080 | <0.001 | 8.02  (7.39 to 8.71) |
| Age in 5 years | 0.028 | <0.001 | 1.03  (1.03 to 1.03) | 0.028 | <0.001 | 1.03  (1.03 to 1.03) |
| Female | -0.266 | <0.001 | 0.77  (0.74 to 0.79) | -0.270 | <0.001 | 0.76  (0.74 to 0.79) |

*IPTW*: Inverse Probability of Treatment/exposure Weight of Capacity utilization for ≥ 85^th^ percentile for daily varying confounders, patient turnover and PCCL (Patient Clinical Complexity Level), The reference categories are (hospital types: university hospitals, Swiss comorbidity weights: <0), The age groups (five-year) are converted into numeric, Clustering of observations by hospital and patient is accounted for both models.

**Table S7.** Adjusted causal effect (total/short term and cumulative daily) of time-varying exposure (capacity utilization) on 14-day in-hospital mortality with truncation of the top and bottom 1% of IPTW

|  | Total/Short term effect with IPTW  (MSM with 1% truncation) | | | Cumulative daily effect with IPTW  (MSM with 1% truncation) | | |
| --- | --- | --- | --- | --- | --- | --- |
|  | Estimate | p value | Causal Odds Ratio (95% CI) | Estimate | p value | Causal Odds Ratio (95% CI) |
| Daily exposure to capacity utilization | | | | | | |
| ≥85^th^ percentile per day | 0.085 | <0.001 | 1.09  (1.04 to 1.13) | 0.012 | <0.01 | 1.01  (1.00 to 1.03) |
| Other adjusted variables | | | | | | |
| Weekend | 0.106 | <0.001 | 1.11  (1.07 to 1.15) | 0.087 | <0.001 | 1.09  (1.05 to 1.13) |
| Hospital types |  |  |  |  |  |  |
| Tertiary care (level 2) | 0.007 | 0.70 | 1.01  (0.97 to 1.05) | 0.007 | 0.70 | 1.01  (0.97 to 1.05) |
| Large basic (level 3) | -0.148 | <0.001 | 0.86  (0.81 to 0.92) | -0.148 | <0.001 | 0.86  (0.81 to 0.92) |
| Medium basic (level 4) | -0.120 | <0.001 | 0.88  (0.83 to 0.95) | -0.119 | <0.001 | 0.89  (0.83 to 0.95) |
| Small basic (level 5) | 0.286 | <0.001 | 1.33  (1.19 to 1.49) | 0.287 | <0.001 | 1.33  (1.19 to 1.49) |
| Elixhauser index (Swiss Comorbidity weights) | | | | | | |
| =0 | 1.092 | <0.001 | 2.98  (2.72 to 3.27) | 1.090 | <0.001 | 2.98  (2.72 to 3.27) |
| >0 to <5 | 0.862 | <0.001 | 2.37  (2.13 to 2.64) | 0.862 | <0.001 | 2.37  (2.13 to 2.64) |
| ≥5 | 2.070 | <0.001 | 7.90  (7.28 to 8.57) | 2.070 | <0.001 | 7.90  (7.28 to 8.56) |
| Age in 5 years | 0.028 | <0.001 | 1.03  (1.03 to 1.03) | 0.028 | <0.001 | 1.03  (1.03 to 1.03) |
| Female | -0.270 | <0.001 | 0.76  (0.74 to 0.79) | -0.270 | <0.001 | 0.76  (0.74 to 0.79) |

*IPTW*: Inverse Probability of Treatment/exposure Weight of Capacity utilization ≥85% for daily varying confounders (patient turnover and PCCL (Patient Clinical Complexity Level), The reference categories are (hospital types: university hospitals, Swiss comorbidity weights: <0), The age groups (five-year) are converted into numeric, Clustering of observations by hospital and patient is accounted for both models.
